# The Market for Bidis, Smokeless Tobacco, and Cigarettes in India: Evidence From Semi-Urban and Rural Areas in Five States

**DOI:** 10.3389/ijph.2021.1604005

**Published:** 2021-05-12

**Authors:** Kevin Welding, Michael Iacobelli, Sejal Saraf, Katherine Clegg Smith, Namrata Puntambekar, Prakash C. Gupta, Joanna E. Cohen

**Affiliations:** ^1^ Department of Health, Behavior and Society, Bloomberg School of Public Health, Johns Hopkins University, Baltimore, MD, United States; ^2^ Institute for Global Tobacco Control, Bloomberg School of Public Health, Johns Hopkins University, Baltimore, MD, United States; ^3^ Healis Sekhsaria Institute for Public Health, Maharashtra, India

**Keywords:** global health, LMICs, illegal tobacco products, price, taxation

## Abstract

**Objectives:** Compare the brand availability, pricing and presence of illicit products in semi-urban and rural areas in India across product types and states.

**Methods:** In late 2017, 382 unique tobacco products were purchased from localities with populations under 50,000 in the states of Assam, Karnataka, Maharashtra, Rajasthan, and Uttar Pradesh. Brand, printed maximum retail price, price paid, tax, and health warning labels (HWLs) were used to compare the market for bidis, smokeless tobacco (SLT), and cigarettes.

**Results:** Brand availability and pricing of SLT products was similar to cigarettes. Brand availability and pricing of bidis was consistent with having many small producers. Bidis and single serving SLT with spice mixtures were more affordable than cigarettes and SLT sold alone. 2% of SLT and 10% of cigarettes did not feature an India HWL.

**Conclusion:** The elimination of single serving SLT packets and the removal of tax exemptions for small producers, often exploited by bidi producers, could reduce their respective affordability. State differences in illegal and illicit products could indicate a greater need for enforcement in some states.

## Introduction

India is the second leading consumer, third largest producer and fifth largest exporter of tobacco products globally [1]. According to the 2016–17 Global Adult Tobacco Survey (GATS), 28.6% (266.8 million) of the adult Indian population currently use some type of tobacco. What differentiates India from other countries is that smokeless tobacco (SLT) use exceeds smoked tobacco use; 21.4% (199.4 million) of adults use SLT and 10.7% (99.5 million) use a smoked form. The number of current cigarette smokers (37.5 million) is surpassed by the number of current bidi (a cheap, unfiltered cigarette made of tobacco flakes wrapped in a tendu or temburni leaf) smokers (71.8 million) [2]. Bidis and SLT are particularly popular outside of urban areas and among people with lower incomes and education [3–7].

Compared to the market for cigarettes, where three companies account for 94% of the market [8], the market for SLT and bidis is relatively more complex [9]. There are many types of SLT products (e.g., Khaini, Zarda, chewing tobacco) available and the top two companies account for only 39% of the market [10]. SLT products are often sold in single-serving packets that are inexpensive and easily accessible [11, 12]. The bidi industry is largely unorganized; firms employ many small-scale, local producers, that are unregulated, with a multitude of small household production units and distributed by as many as a million retailers [13]. The Indian Health Ministry has noted that the bidi industry has taken advantage of the tax concession for small producers by closing large producers and replacing them with small ones under different names [14]. Previous research has shown that bidis are much less expensive than cigarettes [13, 15]. Comparisons of the health warning labels (HWLs) on tobacco products in India show that compliance and printing quality for SLT and bidis is much worse than cigarettes [16–18].

This study focuses on brand availability, tax avoidance, prices relative to the maximum retail price (MRP), and the presence of illegal/illicit tobacco products. Brand availability, both within a state and across states, is important to study as the number of brands for a particular type of product can impact the pricing, the enforcement efforts required, and influence whether state policies can be effective or if country-level policies are necessary. Tobacco producers in India are required by the government to pay taxes and display the statement “inclusive of all taxes” on the packaging, which has the equivalent function of a tax stamp. Tax levels vary by tobacco product with the rate being significantly lower for bidis than cigarettes and SLT. A tax exemption exists for companies producing under two million rupees (30,770 USD) worth of products. Products that do not display “inclusive of all taxes” and are not exempt are illicit as taxes were not paid. Companies are also required to display an MRP, which is intended to avoid price spikes. Tobacco products in India are required to display HWL covering 85 percent of the two principal display areas (e.g., front and back of cigarette pack). Regulations require that tobacco products with older versions of the Indian HWL not be sold following the enactment of a newer HWL policy. Products sold with an old HWL are considered illegal to sell, but not illicit, which is a term designated for products that avoid required taxation (e.g., smuggled foreign products).

Because a majority (68.8%) of the Indian population resides outside urban areas [19] and the consumption of bidis and SLT is predominantly in these settings, this study aimed to examine semi-urban and rural areas. Data from these areas are particularly valuable since studies often focus on major cities. The 2016–2017 GATS compared the expenditure for the last purchase for bidis, SLT, and cigarettes in rural and urban areas. Similar expenditures were found for bidis and SLT, while the amount spent on the last purchase of cigarettes was higher in urban areas [2]. Recent research on the bidi industry focuses on the women workforce [20], the economic impact of bidi smoking [21], and trends in bidi consumption relative to cigarettes [22, 23]. This study goes beyond previous studies and to assess the similarities and differences in the tobacco product markets across product types in the number of brands available, the pricing strategies, and the presence of illicit/illegal products in semi-urban and rural areas in five states in India. Understanding how different tobacco products are marketed and how policies are implemented across product types allows us to understand if policies have unintended impacts on the relative demand for different tobacco products.

## Methods

The Tobacco Pack Surveillance System (TPackSS) systematically collects unique tobacco packs sold in low- and middle-income countries with high tobacco use. The system attempts to collect the Universe of unique tobacco packs in a country by visiting diverse neighborhoods in multiple cities [24]. This study used an adaptation aimed at collecting the Universe of unique tobacco products available in rural and semi-urban areas in five Indian states. In late 2017, bidis, SLT, and cigarettes were collected in Assam, Maharashtra, Karnataka, Rajasthan, and Uttar Pradesh. These states were selected based on geographic diversity and prevalence of bidi and SLT users. The rural population in these five states account for about 40% of the rural population in India [25].

Within each state we identified the top five most populous districts, excluding those containing state capitals. From those districts, we selected two or three based on geographic proximity for pragmatic reasons like travel time between districts.

Within the selected districts, localities were selected from the Census Bureau of India classifications [25]. One locality with population of 20,000–49,999 (class three city), two with populations of 10,000–19,999 (class four towns) and two with populations of 5,000–9,999 (class five villages) were selected for data collection within each district (Supplementary Appendix S1). Within each selected locality, we identified “hubs” (e.g., religious temples, educational structures and post offices), which were proxies for neighborhoods. Between three and five geographically distinct hubs were pre-selected before data collection in each locality. Where available, multiple backup hubs were identified in case a primary hub was incorrect, inaccessible, or a lack of tobacco vendors prevented data collection.

At each hub, a walking protocol was used to construct a sample of tobacco vendors where unique bidis, SLT, and cigarettes were purchased within each state. The types of vendors selected (small grocery stores, paan bidi shops, street vendors, tobacco specialists; Supplementary Appendix S2) were based on the most popular tobacco vendor types according to Euromonitor [26] and GATS [2]. At each vendor visited, any unique presentations not already purchased within a state were collected for each type of tobacco product.

Unique presentations were defined as having at least one difference in a design feature or marketing appeal (e.g., different tobacco quantities, number of sticks, brand presentations, or colors). SLT that was sold with a spice mixture was considered unique if the pairing was unique. HWLs from the same cycle were not considered a unique difference.

The resulting sample was coded independently by two trained coders to capture information about each tobacco package. Analysis included descriptive comparisons of the number of unique product offerings, the number of brands, price, and the type of HWL [current Indian, old Indian (illegal), none/foreign (illicit)] across tobacco products and states.

## Results

Purchases were made at 125 stores (96 small grocery stores, 22 paan bidi shops, five street vendors, two tobacco specialists) across five localities in each state and the sample included 71 bidi packs, 240 SLT packages, and 71 cigarette packs (Table 1). SLT products had the greatest number of unique product offerings available in every state. In Assam, Maharashtra, and Uttar Pradesh, the number of unique cigarette packs were greater than bidi packs, while the opposite was true in Karnataka and Rajasthan. The state samples were similar across tobacco products with a few exceptions. The availability of unique SLT offerings was greater in Uttar Pradesh relative to other states and the availability of unique bidi offerings in Assam and Maharashtra was relatively smaller.

**TABLE 1:** Unique purchases and brands by state and product type (Tobacco Pack Surveillance System (TPackSS), India, 2017).

State	Bidi			SLT			Cigarettes		
	N	Number of unique brands	Average packs per brand	N	Number of unique brands	Average packs per brand	N	Number of unique brands	Average packs per brand
Assam	8	7	1.14	42	22	1.91	11	5	2.20
Karnataka	19	18	1.06	36	16	2.25	8	4	2.00
Maharashtra	8	6	1.33	30	15	2.00	18	11	1.64
Rajasthan	21	17	1.24	42	28	1.50	16	12	1.33
Uttar Pradesh	15	11	1.36	90	39	2.31	18	10	1.80
Combined	71	55	1.29	240	99	2.42	71	29	2.45

Note: SLT = smokeless tobacco.

### Unique Brands

An examination of the number of brands available to consumers for each product type (Table 1) provides a sense of the diversity for each tobacco product market. For example, if we purchased 20 products and found 20 unique brands (average products per brand = 1) this would be a more segmented market than if we purchased 20 products and found 1 unique brand (average products per brand = 20). The bidi collection had the most segmented market with 55 different brands from the 71 bidi packs, an average of 1.29 packs per brand. The average number of packs per bidi brand was lower than SLT and cigarettes in every state. Only three bidi brands (5%) were found in more than one state. SLT had the greatest number of brands available, with 99 different brands from the 240 SLT packets. The average packets per brand (2.42) means that the SLT market was less segmented than the bidi market. Uttar Pradesh had the greatest variety of unique SLT brands (*n* = 39) and the least market segmentatio. SLT products appeared to have a wider market presence with 16 brands (16%) available in multiple states. In each state, the number of unique cigarette packs purchased was closer to the number of bidis than SLT, but the average packs per brand (2.45) indicated a market diversity similar to SLT. Similarly, the percentage of the 29 cigarette brands found in multiple states (24%) was more comparable to the SLT market. Lists of the brands collected by product type can be found in Supplementary Appendices S3–S5.

### Price

The median price for a pack of bidis was 12 rupees (₹) (range:₹5–₹25). The median price for SLT products was ₹5 (range:₹1–₹300). The median price for a pack of cigarettes was ₹95 (range: ₹5–₹300). To make the prices of these products more comparable they were adjusted by the number of sticks for bidis (range:5–25) and cigarettes (range:10–20) and weight per Gram for SLT. For comparison purposes we focus on the 103 SLT only purchases with a weight listed and adjusted for weight (range:0.3–100).

During data collection ₹65 was worth about one US dollar. The median price of a pack of bidis was $0.18 and the per stick median price was $0.01 (range:$0.004–$0.015). The median price of the SLT was $0.08 and the per Gram median price was $0.01 (range:$0.002–$0.27). The median price for a pack of cigarettes was $1.46 and the per stick median price was $0.14 (range:$0.008–$0.26). Similar to the package prices, the per stick bidi price was similar to the per Gram SLT price and the price range for bidis was smaller than SLT and cigarettes. Cigarettes had a higher per unit cost than bidis and SLT but had a similar price range to SLT.

Table 2 provides the comparison between the MRP printed on the packaging and the price paid. Only 6% of bidi purchases had the required MRP printed on the packaging compared to over 90% of SLT and cigarettes. Six of the seven cigarette packs without a printed MRP were packs without an Indian HWL. For the small sample of bidis with printed MRPs, most printed prices matched the price paid. Similarly, the majority (57%) of SLT packs without spice mixtures were bought at their MRP. For SLT packs with spice mixtures there was still a higher frequency of paying the MRP, while cigarette packs were most frequently purchased for more than their printed MRP.

**TABLE 2:** Printed maximum retail price vs price paid (Tobacco Pack Surveillance System (TPackSS), India, 2017).

	Sample size	w/Printed MRP	Over MRP	Equal MRP	Under MRP
Bidis	71	4 (6%)	1 (25%)	3 (75%)	0 (0%)
SLT (w/spice mixture)	121	116 (96%)	44 (38%)	56 (48%)	16 (14%)
SLT (only)	119	112 (94%)	33 (29%)	64 (57%)	15 (13%)
Cigarettes	71	64 (90%)	27 (42%)	25 (39%)	12 (19%)

Note: SLT = smokeless tobacco, MRP = maximum retail price.

**FIGURE 1:**
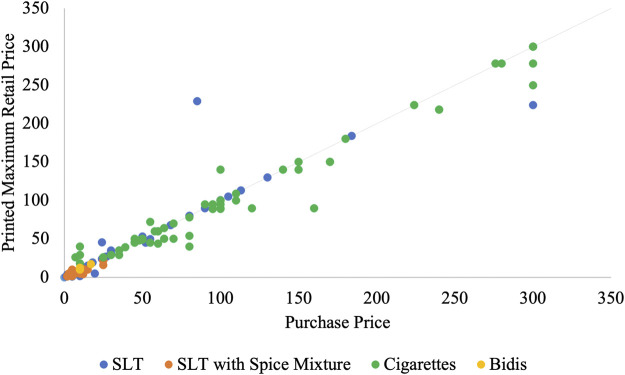
Purchase and printed price, by product type (Tobacco Pack Surveillance System (TPackSS), India, 2017). SLT = smokeless tobacco.

Figure 1 shows combinations of purchase and printed prices for all products that had a printed MRP. The affordability of bidis and SLT with a spice mixture and the wide range of prices for cigarettes and SLT without a spice mixture are evident. The points above the 45-degree line represent products that were bought for less than the MRP, while points below the 45-degree line cost more than the MRP. The purchases in the under MRP category were, on average, 33% lower than the MRP for cigarettes, while only 18% and 19% lower for SLT alone and SLT with spice mixtures, respectively. While cigarettes were more likely to be in the over MRP category, the margin of overpayment was only 20% more than the MRP compared to 116% for SLT alone and 55% for SLT with spice mixtures.

We also examined the frequency that products did not include the statement “inclusive of all taxes”. Unsurprisingly, none of the products without an Indian HWL included the statement indicating that taxes were paid. None of the bidis had the tax statement. SLT and cigarettes with the current HWL included the tax statement at a higher rate than purchases with an older Indian HWL (39 vs. 7% and 100 vs. 89%, respectively).

### Illicit/Illegal Tobacco Products

The HWLs found in India during this data collection can be broadly categorized into three groups: current Indian HWLs, old Indian HWLs, and foreign/no HWLs (Supplementary Appendix S6). The current (2017–18) Indian HWLs were required at the time of data collection. The old Indian HWLs included the “last Indian HWL” required in 2016–17 and “older Indian HWLs” that pre-date the 85% coverage requirement. Products with these HWLs were illegal at the time of data collection. Products not intended for sale in India, with a foreign HWL or no HWL, are illicit.

For bidis, all the collected packs had an Indian HWL on them, but 55% were illegal with an old Indian HWL (Tables 3–5). Similarly, the SLT products collected predominately featured an Indian HWL, but 43% were illegal and 2% were illicit. The collection of cigarettes featured the lowest proportion of illegal packs (25%), but also the highest proportion of illicit packs (10%).

**TABLE 3:** Health warning label distribution by state, bidi (Tobacco Pack Surveillance System (TPackSS), India, 2017).

State	Total	Current (*n*, %)	Last (*n*, %)	Older (*n*, %)	Foreign/None (*n*, %)
Assam	8	2, 25	2, 25	4, 50	0, 0
Karnataka	19	4, 21	13, 68	2, 11	0, 0
Maharashtra	8	3, 38	2, 25	3, 38	0, 0
Rajasthan	21	16, 76	5, 24	0, 0	0, 0
Uttar Pradesh	15	7, 47	4, 27	4, 27	0, 0
Combined	71	32, 45	26, 37	13, 18	0, 0

**TABLE 4:** Health warning label distribution by state, smokeless tobacco (Tobacco Pack Surveillance System (TPackSS), India, 2017).

State	Total	Current (*n*, %)	Last (*n*, %)	Older (*n*, %)	Foreign/None (*n*, %)
Assam	42	22, 52	15, 36	5, 12	0, 0
Karnataka	36	21, 58	13, 36	2, 6	0, 0
Maharashtra	30	20, 67	9, 30	1, 3	0, 0
Rajasthan	42	31, 74	7, 17	3, 7	1, 2
Uttar Pradesh	90	39, 43	24, 27	24, 27	3, 3
Combined	240	133, 55	68, 28	35, 15	4, 2

**TABLE 5:** Health warning label distribution by state, cigarettes (Tobacco Pack Surveillance System (TPackSS), India, 2017).

State	Total	Current (*n*, %)	Last (*n*, %)	Older (*n*, %)	Foreign/None (*n*, %)
Assam	11	10, 91	1, 9	0, 0	0, 0
Karnataka	8	7, 88	0, 0	1, 13	0, 0
Maharashtra	18	9, 50	2, 11	3, 17	4, 22
Rajasthan	16	11, 69	2, 13	2, 13	1, 6
Uttar Pradesh	18	9, 50	5, 28	2, 11	2, 11
Combined	71	46, 65	10, 14	8, 11	7, 10

There are some state-level differences in the HWL distribution for bidis. Rajasthan had the lowest proportion of bidis with an illegal HWL (24%) and Karnataka had the highest proportion (79%). The state-level differences for SLT are less pronounced than bidis. Similarly, Rajasthan had the lowest proportion (24%) of SLT with an illegal HWL, while Uttar Pradesh had the highest (54%). Uttar Pradesh also was one of two states with Rajasthan where we found illicit SLT products.

In every state at least half of the cigarettes purchased had a current HWL. Assam (9%) and Karnataka (13%) had the lowest proportions of illegal HWLs and neither had any illicit packs. The highest proportion of illegal HWLs on cigarettes (39%) was found in Uttar Pradesh, while the highest proportion of illicit packs (22%) was found in Maharashtra. Illicit cigarette packs were also found in Uttar Pradesh (*n* = 2, 11%) and Rajasthan (*n* = 1, 6%).

## Discussion

SLT products had the greatest number of unique product offerings available in every state. This is understandable considering the variety of SLT product types and spice mixtures. For SLT products without spice mixtures, the product offerings were still higher than bidis and cigarettes in Assam and Uttar Pradesh. SLT products were particularly prevalent in Uttar Pradesh, which could be explained by its larger population or greater number of unique brands available. The number of product offerings for bidis are more similar to the product offerings of cigarettes in all states.

Despite the strengths of this study, which include a collection of bidis, SLT, and cigarettes in difficult to study rural and semi-urban areas in five Indian states, there are limitations. The sample of products does not represent the market share of brands, as only one unique presentation of each tobacco product was purchased. Among the products with an 85% HWL, only the first unique presentation was purchased in each state. Given that the sample was intended to capture the breadth of available products, there is more confidence in the price range than the average. Despite the attempt to collect the Universe of tobacco products in each state, given the data collection undertaken, it is possible that some available products were not collected. The purchase of unique products within each state means that there could be some duplicates when presenting aggregated results combined across states. The collection of products in rural and semi-urban areas may not be representative of other rural and semi-urban areas in the same state, or in other states in India. The results may not reflect brand availability and prices in urban areas. For example, we would expect fewer available brands in areas with lower demand for certain products. Previous work on SLT HWL compliance found lower compliance for warning location and warning label elements outside of urban areas [17, 27]. This difference and the finding from the 2016–2017 GATS that participants notice HWLs at lower rates in rural areas for all tobacco products [2] implies that urban areas might have better implementation or stricter enforcement of new policies.

The examination of brand availability revealed that the bidi collection had the smallest average number of products per brand, which was true across states. The average number of products per brand for SLT products was similar to that of cigarettes. Differences in products per brand across state could indicate differences in consumer demand or producer supply for certain tobacco products in these states. The bidi collection also had the smallest number of brands found in more than one state, while the number of SLT brands in more than one state was again similar to that of cigarettes. These findings are consistent with the notion that the bidi industry is highly localized with a movement from large production units to smaller producers.

Bidis and SLT tend to be more affordable than cigarettes. The price ranges for bidis and SLT with a spice mixture are much smaller than the price ranges for cigarettes and SLT without a spice mixture. The lower prices for bidis and SLT could reflect lower production costs or more industry competition, while the larger price ranges for SLT and cigarettes could be indicative of the number of different types and weights of SLT and the perceived differences in quality between economy and premium cigarettes. Bidis were less likely to have a printed maximum retail price on the packaging compared to cigarettes and SLT products and no bidis included the statement “inclusive of all taxes” on packaging. The latter is consistent with the previously mentioned notion that the bidi industry features small-scale production and the presence of a tax exemption for production under two million rupees (30,770 USD). Tax statements were more prevalent on cigarettes than SLT, which could be the result of smaller producers or less enforcement for SLT products. Given the production processes of these tobacco products, it is likely that the bidi industry, which often centers on in-home production, is better suited to take advantage of the tax exemption for small-scale producers.

The examination of the HWLs on tobacco products provided insight about illegal (old Indian HWL) and illicit (no Indian HWL) products. Bidis did the best with no illicit HWLs found, while 2% of SLT products and 10% of cigarette packs did not have an Indian HWL. This result is consistent with lower levels of foreign production for bidis and SLT compared to cigarettes. The proportion of products with old Indian HWLs was lower for cigarettes than SLT products and bidis. This finding could be the result of cigarette inventory turning over faster than bidis and SLT. It is also possible that more companies in the bidi and SLT industries are less likely to implement new policies and more willing to take a chance of being caught. The most noticeable state-level difference was the low proportion of bidis with an old HWL in Rajasthan. This may be an indication of a state-level effect, as opposed to something specific about bidis in Rajasthan, since the percentage of SLT and cigarettes without the current Indian HWL were also low.

This study has important policy implications. At the product level, consistent with previous research, we find that the bidi market is different than the cigarette and SLT markets. Bidi results are consistent with more localized production and distribution, which could require a more extensive effort to inform small-scale bidi manufacturers about how to implement updated policies and regulations and a more extensive enforcement effort to ensure compliance. This is one possible explanation for why a majority of bidis do not feature the current Indian HWL. While there is a range of prices for SLT products and a clear pathway to reduce affordability by eliminating the sale of single serving packets, all bidis were uniformly affordable. The removal of tax exemptions for small-scale producers, which appears to be concentrated among bidi producers, could change the relative affordability. State differences in product offerings could be indicative of differences in consumer demand or available producers, but differences in illegal and illicit tobacco products could indicate a greater need for some states to better enforce country-level policies.

In summation, the price and brand availability across the states of SLT products looked more similar to cigarettes than bidis. The observed brand availability and pricing of bidis is consistent with having many small, local producers. Very few bidis and SLT products were found without an Indian HWL, but both products were more likely to feature old Indian HWLs than the cigarette packs collected. It is important for all countries with more than one tobacco product available to consider how policies could change the relative landscape of the tobacco market. The prevalence of older HWLs and little evidence of the taxation of bidis highlights the implementation, enforcement and policy limitations around India’s most popular smoked tobacco product. Given that all tobacco products are harmful it is important for governments make a concerted effort to disseminate information about new policies widely, enforce requirements across all segments of the industry, and close policy loopholes that appear to be more easily exploitable for one product than the rest.
