# Supplementary material for: The Market for Bidis, Smokeless Tobacco, and Cigarettes in India: Evidence From Semi-Urban and Rural Areas in Five States
Source: Int J Public Health. 2021 May 12;66:1604005. doi: 10.3389/ijph.2021.1604005 (PMC8284861; doi:10.3389/ijph.2021.1604005)
Supplement: Supplementary file 1 [file Table1.docx]

**SUPPLEMENTAL APPENDIX**

**Appendix S1:** Data collection map (Tobacco Pack Surveillance System (TPackSS), India, 2017).


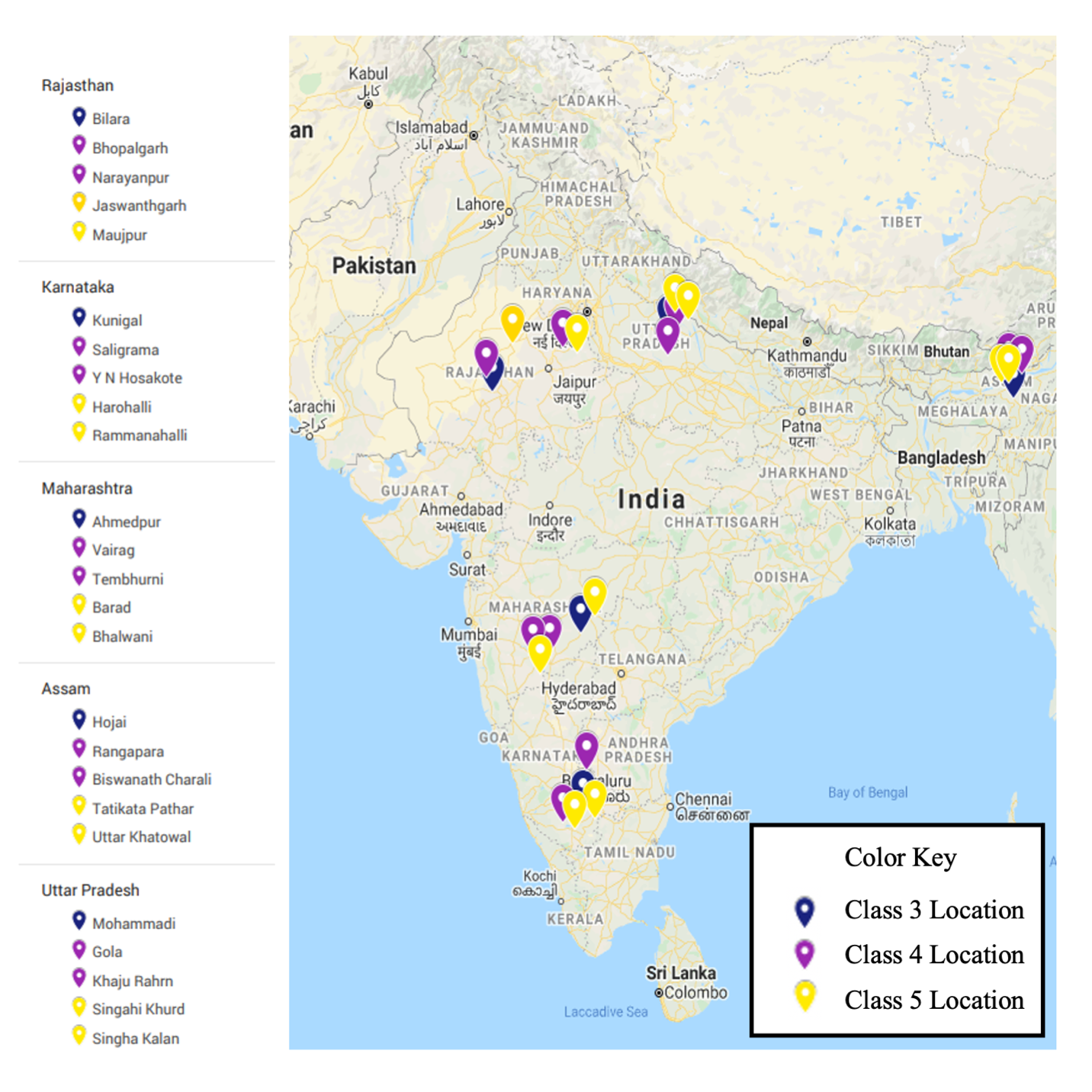


**Appendix S2:** Tobacco vendor definitions (Tobacco Pack Surveillance System (TPackSS), India, 2017).

| **Type of Vendor** | **Definition** |
| --- | --- |
| **Independent Small Grocer** | Small, one-person establishment that sells a limited variety of goods, including cigarettes, cleaning supplies, and food staples. |
| **Kiosk/Paan Bidi Shop** | A small enclosed structure, often freestanding, open on one side or with a window, used as a booth to sell cigarettes, bidis, and paan products. |
| **Street Vendor** | A retail salesperson without an established place of business. A person, or persons, travelling on public streets, public sidewalks, public property, or private and carrying, conveying or transporting items such as food, beverages, flowers, and balloons, offering and exposing the same for sale by hand or from a mobile type device such as push cart. |
| **Tobacco Specialist** | Store specializing in all manners of tobacco products. |

| **State** | **Assam** | **Karnataka** | **Maharashtra** | **Rajasthan** | **Uttar Pradesh** |
| --- | --- | --- | --- | --- | --- |
| **Number of Unique Packs Collected** | 8 | 19 | 8 | 21 | 15 |
| **Number of Unique Brands** | 7 | 18 | 6 | 17 | 11 |
|  |  |  |  |  |  |
| **Brand Names** | Babu | Ambika | Ghoda | Anchor | Abdul Sattar No.321 |
|  | Devdas | Bharath | Govind | Bharat | Baba |
|  | Dumdum | Hajjaj | Mangalore Ganesh | Bharath | Badi |
|  | Kissan | J. M. Amar Deep | Moon Mark Jadi | Desai | Balak |
|  | Mother India | Jagannath | Munsi | Golden | Bulbul |
|  | Mukta | K. M. Peer | Sambhaji | Haribhai | Mangalore Ganesh |
|  | Sankha | Kunigal New National |  | Howrah | Orissa |
|  |  | Madhugiri.S.Janata |  | Jaswant | Pataka |
|  |  | Mangalore Bazar |  | Jhanda | Patel 95 |
|  |  | Mangalore Ganesh |  | Jiyo | Pooja |
|  |  | Mayura |  | Mama No.102 | Sher |
|  |  | Mysore Lakshmi |  | Manu |  |
|  |  | New Raja |  | O2 |  |
|  |  | New Seetha |  | Pakiz |  |
|  |  | Noor |  | Pataka |  |
|  |  | S. K. |  | Unta |  |
|  |  | Special Lakshmi |  | Vikram |  |
|  |  | Sri Lakshmi |  |  |  |

**Appendix S3:** Bidi brands, by state (Tobacco Pack Surveillance System (TPackSS), India, 2017).

**Appendix S4:** Smokeless tobacco brands, by state (Tobacco Pack Surveillance System (TPackSS), India, 2017).

| **State** | **Assam** | **Karnataka** | **Maharashtra** | **Rajasthan** | **Uttar Pradesh** |
| --- | --- | --- | --- | --- | --- |
| **Number of Unique Packs Collected** | 42 | 36 | 30 | 42 | 90 |
| **Number of Unique Brands** | 22 | 16 | 15 | 29 | 39 |
|  |  |  |  |  |  |
| **Brand Names** | Ashirbad | Badshaha | Bhajki Masheri | Agrawal | Bangali tobacco |
|  | Baba | Chaini | Chaini | Asli more | Chand Tara Marka |
|  | Bandar | Chetna | G-1 | Baghban | Chunnu |
|  | Bengali | Hans | Gai | BGM | Dabangg |
|  | Bhagat | Madhu | Harikrishna Niwas | BHR | Dhameja |
|  | Chand Tara Marka | Mirage | Ipco | DB | Gagan |
|  | Gopal 132 | Parag | Kisan Tota | Ganesh | Gauraiya |
|  | Jahaz Marka | RMD | Krishan Chhap | Himalaya | Gopal 132 |
|  | K | Royal | Mirage | Horse Brand | Hanuman Chhap |
|  | Kaka Chhap | S1 | Om Pandharpuri | JZ | Har Singar |
|  | Kanchann | S99 | Ratna | Kuber | Hathi Gola |
|  | KP | Shanti | RMD | Maha Pasand | Horse Brand |
|  | Nevla | STR-1 | S99 | Mirage | Jai Ho |
|  | R-R | Umbrella | Surya | MJ | Kakku |
|  | Raja | V-1 | V-1 | N-1 | KP |
|  | S-TEN | Vani |  | N-11 | Kuber |
|  | SH 001 |  |  | Natraj | Megha Shree |
|  | Shahi |  |  | Nazar | Musa |
|  | SR-1 |  |  | Nirala | N-1 |
|  | SVS |  |  | NT-1 | N-11 |
|  | Tulsi |  |  | Raja | Palang Tore |
|  | V-1 |  |  | Saat Taarey | Pukar |
|  |  |  |  | Shankar | Radhe Shahi |
|  |  |  |  | Sugandh Sagar | Ratna |
|  |  |  |  | Sugandhar | Ravi |
|  |  |  |  | T0 | Royal |
|  |  |  |  | Tulsi | Rupa |
|  |  |  |  | V-1 | Saburi |
|  |  |  |  | Z Gold | Safal |
|  |  |  |  |  | Sangam |
|  |  |  |  |  | Sanghini |
|  |  |  |  |  | Saroj |
|  |  |  |  |  | Shahi |
|  |  |  |  |  | Shayamal |
|  |  |  |  |  | Sukka |
|  |  |  |  |  | T0 |
|  |  |  |  |  | Tulsi |
|  |  |  |  |  | V-1 |
|  |  |  |  |  | Z Gold |

**Appendix S5:** Cigarette brands, by state (Tobacco Pack Surveillance System (TPackSS), India, 2017).

| **State** | Assam | Karnataka | Maharashtra | Rajasthan | Uttar Pradesh |
| --- | --- | --- | --- | --- | --- |
| **Number of Unique Packs Collected** | 11 | 8 | 18 | 16 | 18 |
| **Number of Unique Brands** | 5 | 4 | 11 | 12 | 10 |
|  |  |  |  |  |  |
| **Brand Names** | Charms | Berkeley | Attack 10's | B10 | Capstan |
|  | Gold Flake | Flake | Bristol | Cavanders | Classic |
|  | Hitler Black | Gold Flake | Charminar | Four Square | E-10 |
|  | Navy Cut | Navy Cut | Classic | Gold Flake | Gold Flake |
|  | Wills |  | Djarum Black | Hitler Black | Gudang Garam |
|  |  |  | Dunhill | Perfect | Khukuri |
|  |  |  | Gold Flake | Player's | Navy Cut |
|  |  |  | Gudang Garam | Shooter | P10 |
|  |  |  | More | Ten10 | Total |
|  |  |  | Navy Cut | Total | Wills |
|  |  |  | One&Only | Wills |  |
|  |  |  |  | Win |  |

**Appendix S6:** Example images of health warning labels, by category (Tobacco Pack Surveillance System (TPackSS), India, 2017).

| Current Indian HWLs | Old Indian HWLs | | Foreign/No  HWLs |
| --- | --- | --- | --- |
|  | Last Indian  HWLs | Older Indian  HWLs |  |
| 85% Indian HWLs that were required during the time of data collection (2017-18) | 85% Indian HWLs that were required in 2016-17 before the data collection | Indian HWLs before the increase to 85% coverage. | HWLs that are not from India. |
| 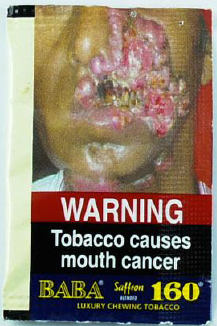 | 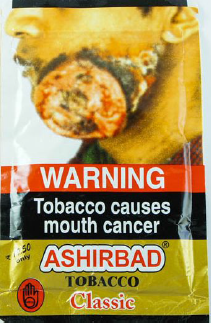 | 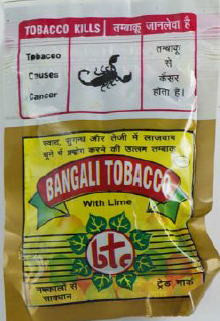 | 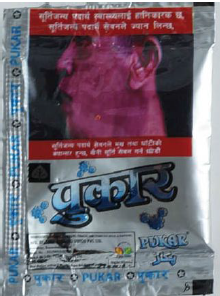 |

Note: HWLs=health warning labels.
